# Established breast cancer stem cell markers do not correlate with *in vivo* tumorigenicity of tumor-initiating cells

**DOI:** 10.3892/ijo.2012.1654

**Published:** 2012-10-05

**Authors:** CHRISTIAN LEHMANN, GABRIELE JOBS, MARKUS THOMAS, HELMUT BURTSCHER, MANFRED KUBBIES

**Affiliations:** Discovery Oncology, Roche Diagnostics GmbH, D-82377 Penzberg, Germany

**Keywords:** breast cancer, stem cells, tumor initiating cells, mammospheres

## Abstract

The tumor-initiating capacity of primary human breast cancer cells is maintained *in vitro* by culturing these cells as spheres/aggregates. Inoculation of small cell numbers derived from these non-adherent cultures leads to rapid xenograft tumor formation in mice. Accordingly, injection of more differentiated monolayer cells derived from spheres results in significantly decelerated tumor growth. For our study, two breast cancer cell lines were generated from primary tumors and cultured as mammospheres or as their adherent counterparts. We examined the *in vivo* tumorigenicity of these cells by injecting serial dilutions into immunodeficient mice. Inoculation of 10^6^ cells per mouse led to rapid tumor formation, irrespective of cell line or culture conditions. However, after injection of only 10^3^ cells, solely sphere cells were highly tumorigenic. *In vitro*, we investigated differentiation markers, established breast CSC markers and conducted mRNA profiling. Cytokeratin 5 and 18 were increased in both monolayer cell types, indicating a more differentiated phenotype. All cell lines were CD24^−^/CD44^+^ and did not express CD133, CD326 or E-cadherin. ALDH1 activity was not detectable in any cell line. A verapamil-sensitive Hoechst side population was present in sphere cells, but there was no correlation with tumorigenicity *in vivo*. mRNA profiling did not reveal upregulation of relevant transcription factors. *In vitro* cell cycle kinetics and *in vivo* tumor doubling times displayed no difference between sphere and monolayer cultures. Our data indicate that intrinsic genetic and functional markers investigated are not indicative of the *in vivo* tumori-genicity of putative breast tumor-initiating cells.

## Introduction

Genetic and epigenetic diversity as well as physiological variations due to intratumoral environmental heterogeneity is a hallmark of solid tumors *in vivo*(1,2). Recently, this heterogeneity became more complex by the identification of a putative cancer stem cell (CSC) or tumor-initiating cell (TIC) population. These cells were shown to exhibit epithelial-to-mesenchymal-transition (EMT) characteristics and seem to be more aggressive (3,4). However, there is no firm evidence whether these populations truly exist in solid cancers (5,6) and unique phenotypic or physiological TIC markers are not identified yet (7–9).

In breast cancer, the *in vivo* inoculation of low cell numbers of CD24^−^/CD44^+^ but not CD24^+^/CD44^+^ or ESA-purified cells from primary tumors gave rise to xenograft tumors (10). The relevance of this marker combination has been confirmed for breast tumor cells lines, although the percentage of putative CD24^−^/CD44^+^ breast cancer tumorsphere TICs did not correlate with tumorigenicity (11). Furthermore, the molecular and phenotypic analysis of primary invasive breast carcinomas revealed that CD24^−^ and CD24^+^ subpopulations were present but this could not be correlated to any tumor characteristic (12,13). Moreover, CD24^+^ cells were found to be significantly increased in distant metastases and readily give rise to invasive progeny, questioning the relevance of CD24 expression as an indicator of TICs (12,14).

Current experimental evidence indicates that the CD24-population might represent a more drug resistant phenotype. In several *in vitro* breast cancer models, the CD24^+^/CD44^+^ population declined but the CD24^−^/CD44^+^ fraction increased after herceptin treatment (15). In a similar study, paclitaxel treated CD24^−^/CD44^+^ breast cancer cells were more resistant to cytotoxic drug treatment compared to the total population (16). In line with these studies, a CD24^−^/CD44^+^ gene expression signature was reported in breast cancer patients after chemo- or endocrine therapy (17). However, controversial data are reported for drug treated breast cancer patients. For example, a histochemical analysis of breast carcinomas revealed a lower percentage of CD24^−^/CD44^+^ cells after chemotherapy and there was no correlation with chemotherapy response or patient survival (18).

In addition to cell surface markers, functional parameters such as aldehyde dehydrogenase activity (ALDH1) or the presence of an ABC transporter dependent Hoechst side population (SP) were suggested to identify breast TICs. In a variety of breast cancer cell lines, only the ALDH1 positive cell fraction developed xenograft tumors (19–21) and lower metastasis-free survival correlated with increased ALDH1 expression in inflammatory breast cancer (21). Furthermore, in established breast cancer cell lines the Hoechst SP was shown to be more resistant to paclitaxel treatment and ionizing radiation and displayed a higher *in vivo* take rate (16,22). Additionally, a genetic analysis indicated that the tumor-initiating cellular phenotype with EMT characteristics was regulated by transcription factors like Twist, Snail or Zeb (4,23). In breast cancer cells, Twist expression correlates with an increase of TIC parameters such as CD24^−^/CD44^+^ expression, enhanced ALDH1 activity and a higher SP fraction (24). Further support is given by Twist or Snail expressing, immortalized human mammary epithelial cells acquiring EMT characteristics and a more tumorigenic phenotype *in vivo*(25).

Besides the serial transplantation of tumors, the mammo- or tumorsphere technology is widely accepted for the cultivation of mammary stem- or tumor-initiating cells (26). Pluripotency and differentiation capability of TIC spheres was indicated by the induction of differentiation related markers such as cytokeratins in their monolayer derivatives (11,27).

Investigating CD marker expression, ALDH1 activity and SP fraction, we report that no difference between highly and low tumorigenic cells was observed. Also, stem cell relevant transcription factors were not increased in the highly tumorigenic spheres derived from cell line S2N. Hence, we suggest that still unknown tumor cell markers and/or environmental factors affect the increased tumorigenicity of sphere cells *in vivo*.

## Materials and methods

### Tumor specimens and cells

Tumor tissue from breast cancer patients (no.1 female, 55 years, G2; no.2 female, 85 years, G2) was obtained at surgical treatment, in accordance with the ethical standards of the responsible institutional committee at the University of Palermo on human experimentation. Diagnosis was based on the histological analysis and involvement of regional lymph nodes. Staging was established according to the UICC TNM classification of malignant tumor.

Enzymatic dissociation was performed using collagenase (1.5 mg/ml, Aldrich, Taufkirchen, Germany) in PBS for 1 h at 37°C. Freshly purified breast tumor cells were depleted of erythrocytes and leukocytes by ammonium chloride lysis and microbeads, respectively (Miltenyi Biotec, Bergisch-Gladbach, Germany). Mammospheres were grown as described previously in mammary epithelial basal medium (Lonza, Cologne, Germany) utilizing T75 low adhesion cell culture flasks (Corning Life Sciences, Wiesbaden, Germany) and passaged every 3 to 4 days after sphere formation (26). Monolayer cultures were derived from mammosphere cells and cultivated in collagen coated T75 cell culture flasks (BD Biosciences) using mammary epithelial basal medium supplemented with 10% FBS (Invitrogen). Monolayer cells were allowed to differentiate for at least 10 days prior to analysis.

### Cell cycle kinetics

Cell cycle kinetic analyses were performed as described previously (28). DNA fluorescence of nuclei was recorded using a FACS LSR II instrument (BD Biosciences). Cell aggregates were excluded by PI-W/PI-A gating.

### Cell surface marker analysis

Disaggregated sphere or monolayer cells (2×10^5^) were suspended in 100 *μ*l ice-cold MEBM/10% FBS. CD24-PE (clone ML5, BD Biosciences), CD29-A488 (clone TS2/16, BioLegend, Eching, Germany), CD44-FITC (clone G44-26, BD Biosciences), CD133-PE (clone TMP4, eBioscience), CD324 E-cadherin-A647 (clone 67A4, BioLegend), CD326 EpCAM-A647 (clone 1B7, eBioscience) antibodies and appropriate mouse-isotype controls (BD Biosciences, BioLegend) were added according to the manufacturer’s recommendations. Cells were stained for 30 min at 4°C in the dark and washed twice with 250 *μ*l MEBM/10% FBS.

### Aldehyde-dehydrogenase-1 and side population analysis

ALDH1 activity was measured using the AldeFluor assay kit (Aldagen). Briefly, 5×10^5^ cells were suspended in 500 *μ*l assay buffer containing 2.5 *μ*g ALDH1 substrate (BAAA) and incubated for 30 min at 37°C. An additional sample was incubated concurrently with the specific ALDH1 inhibitor diethylaminobenzaldeyde (DEAB).

The SP analysis was performed with 1×10^6^ cells/ml resuspended in 500 *μ*l pre-warmed medium containing 5 *μ*g/ml Hoechst 33342 (Sigma) in the presence (100 *μ*g/ml) or absence of the ABCG2 inhibitor verapamil (Sigma). Samples were incubated for 90 min at 37°C. Subsequently, cells were pelleted at 375 g for 6 min at 4°C, resuspended in 300 *μ*l ice-cold medium and kept on ice until flow cytometric analysis.

### Flow cytometry

Cells were analyzed on a FACSCanto II or LSR II (BD Biosciences). Appropriate lasers and filters were used for PE, FITC, A647 and A488 fluorescence recording. Hoechst 33342 excitation was performed at 355 nm and emission analysis was done utilizing a 695/40 (Hoechst Red) and a 450/20 band pass filter (Hoechst Blue). PI was excited at 488 nm and emission recording was done with a 630/30 filter. Viable cells were identified as PI-negative (2 *μ*g/ml). Aggregates were excluded by single cell gating in the side scatter/forward scatter width plot in all experiments. Flow cytometry data were analyzed with the BD FACSDiva (BD Biosciences) or FlowJo software (TreeStar, Olten, Switzerland).

### Protein isolation and western blot analysis

Western blot analysis was carried out using the NuPAGE System (Invitrogen) applying cells (2×10^5^/lane) lysed in RIPA buffer (Sigma), supplemented with protease inhibitor cocktail (Roche Diagnostics, Mannheim, Germany). For identification of relevant proteins, PVDF membranes were incubated with 1:1,000 dilutions of rabbit polyclonal anti-human cytokeratin 5 (CK5; Abcam, Cambridge, UK), mouse monoclonal anti-human CK18 (clone C-04; Biozol, Eching, Germany), mouse monoclonal anti-human vimentin (clone V9; Abcam) or mouse monoclonal anti-human GAPDH (clone 6C5; Millipore, Schwalbach, Germany) at 4°C overnight. The membranes were then washed, incubated with POD-conjugated anti-mouse or anti-rabbit secondary antibody (Roche) at a dilution of 1:1,000 and washed again. Bound secondary antibodies were detected using standard chemiluminescence protocols utilizing Lumi-Light western blotting substrates and Lumi-Film detection film (Roche).

### Gene expression analysis

Total-RNA was isolated using an RNeasy mini kit (Qiagen, Hilden, Germany) followed by cDNA synthesis utilizing a cDNA synthesis kit (Roche). Double-stranded cDNA was purified with a Microarray Target Purification Kit (Roche) and transcribed into cRNA using the Roche Microarray RNA Target Synthesis Kit (T7). cRNA was purified with RNeasy Mini-Spin Columns (Qiagen). All kit procedures were performed according to the manufacturer’s instructions.

A total of 20 *μ*g of the purified cRNA were fragmented and processed for hybridization to Human Genome U133 Plus 2.0 arrays and scanning with an Affymetrix Gene Chip Scanner 3000 (7G) according to Affymetrix protocols. All samples were measured in triplicates. Data were analyzed using in-house software or Partek analysis suite (Partek).

### Tumor xenograft mouse studies

Female NOD/SCID mice were purchased from 2 M&B Bomholtgard (Ry, Denmark). Animals were quarantined for one week for acclimatization and observation. Animals were kept under SPF-conditions according to the international guidelines (GV-Solas; Felasa; TierschG). All experiments were reviewed and approved by the local government (Regierung von Oberbayern; registration no. 211.2531.2-22/2003). 1×10^3^, 1×10^4^ or 1×10^6^ tumor cells (viability >90%) per 50 *μ*l Matrigel (BD Biosciences) were injected subcutaneously. Sphere cells were inoculated into the right and monolayer cells into the left flank of the mice. Inoculation was directed to the backbone of the mice. Tumor growth from 10 mice in each cohort was quantified by caliper measurements. Tumor volume was calculated by callipering the largest diameter (A) and its perpendicular (B) according to the NCI protocol [TV = (A × B^2^)/2].

## Results

### In vivo, tumor-initiating cell characteristics are only present in S2N spheres, but not in S2N monolayer cells or S2 spheres or monolayer cells

Tumor xenograft growth after inoculation of low cell numbers in mice is an important criterion for cancer stem cells *in vivo*. We therefore verified the tumor-initiating cell capacities of our cell lines by inoculating serial dilutions of disaggregated cells from monolayer and sphere cultures. The cell lines S2 and S2N were isolated from human breast cancer tumors and either cultivated on tissue culture plates (to differentiate) or in suspension (to remain undifferentiated). These putative breast cancer sphere TICs were subcutaneously inoculated into the right flank whereas breast cancer monolayer cells were inoculated into the left flank of the mice.

**Figure 1:**
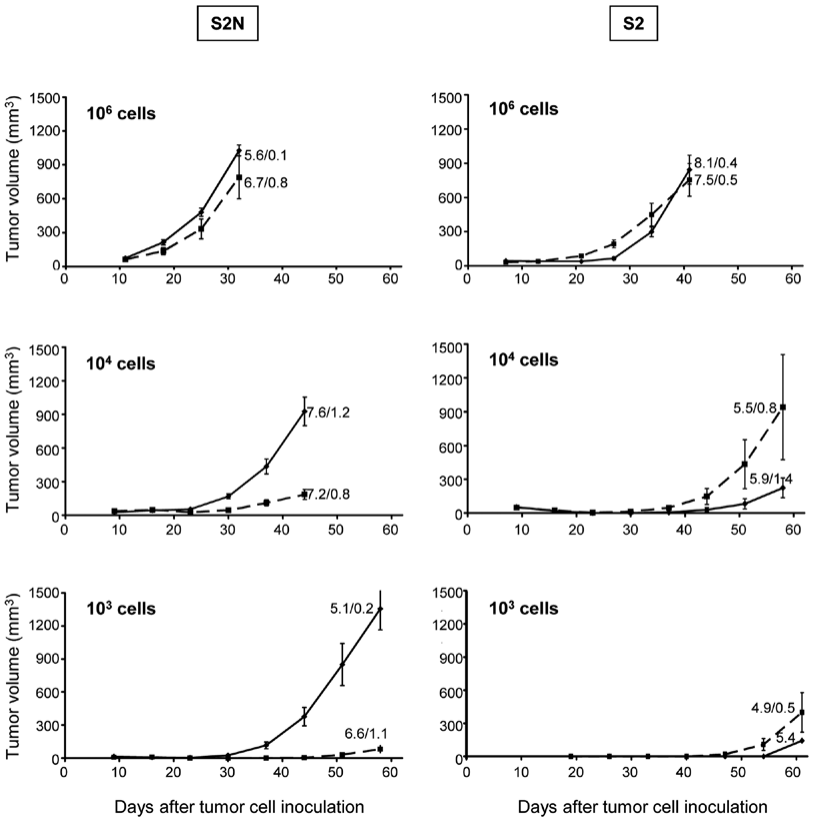
Tumor xenograft growth characteristics of S2N and S2 breast cancer sphere and monolayer cells at different cell number inoculations. Subcutaneous injection of sphere cells into the right flank (solid lines) and monolayer cells into the left flank (broken lines), respectively. Tumor size values represent mean ± SEM (10 mice per group). The numbers adjacent to the lines indicate the mean ± SEM of the population doubling times calculated from the xenograft growth curves (S2 sphere 10^3^ group: tumor growth only in 1/10 mice).

As shown in Fig. 1, there were no differences in tumor growth between S2 spheres and their respective monolayer cells after inoculation of 10^6^ cells.

At lower inoculation cell numbers of 10^4^, we observed an even increased monolayer cell derived xenograft tumor growth which, however, was due to the exceptionally high growth rate of 3 tumors in the study cohort of this experiment (Fig. 1 right column). Poor *in vivo* growth was evident in the S2 10^3^ cell inoculation group for monolayer as well as sphere cells. S2N sphere or monolayer cells inoculated with 10^6^ cells displayed growth characteristics comparable to S2 cells. However, there was an increasingly strong and significant delay in tumor growth of monolayer cultures compared to sphere cultures when the inoculation cell numbers were reduced to 10^4^ (p<0.01 at all study days). The reduced tumor growth was even more pronounced at 10^3^ cells (Fig. 1 left column; p<0.01 at all study days).

In order to see whether individual growth rate differences account for the different xenograft growth kinetics, we calculated the population doubling times from the *in vivo* growth curves. As evident by the numbers in Fig. 1, the population doubling times of xenograft tumors ranged from 4.9 to 8.1 days; however, there was no significant alteration between different cell numbers. Furthermore, *in vitro* population doubling times between sphere and monolayer cells as well as the highly tumorigenic S2N and weakly tumorigenic S2 cell model were similar (data not shown).

### Cell morphologies and cell cycle kinetics of highly and weakly tumorigenic cell lines are similar

**Figure 2:**
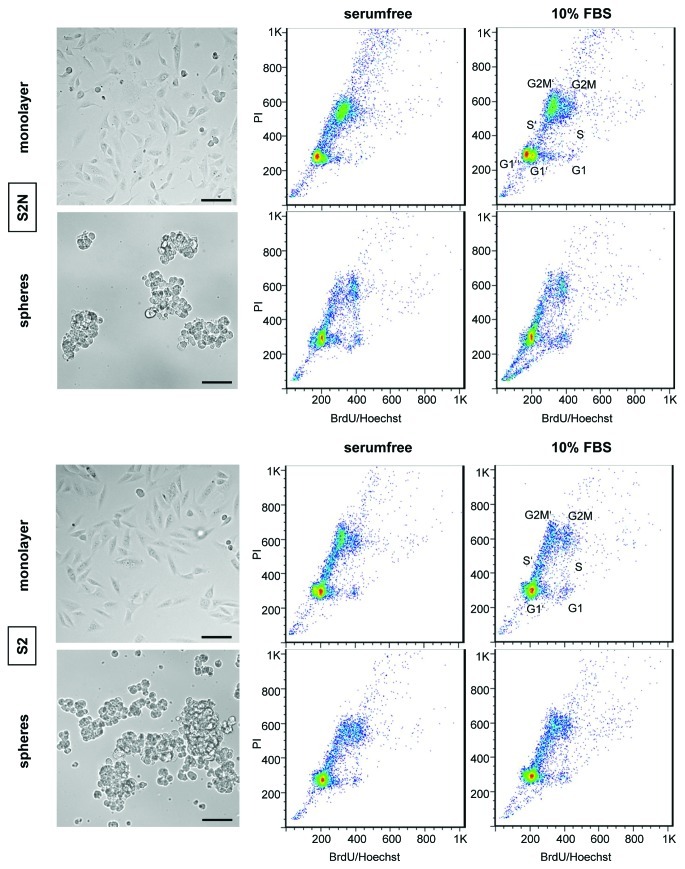
Morphology and cell cycle kinetics of monolayer and sphere S2N and S2 breast cancer cells cultivated under serum-free FGF/EGF and 10% FBS cell culture conditions. Bright field microscopic images of more spindle-like monolayer and aggregated sphere cells [Axiovert 135 microscope (Zeiss), CoolSnap K4 camera (Visitron), bar length 100 *μ*m]. The BrdU/Hoechst FACS cell cycle kinetic analysis was done after a 48 h BrdU labeling period (1st cell cycle, G1, S, G2M; 2nd cell cycle, G1’, S’, G2M’; 3rd cell cycle G1”).

Both the highly tumorigenic S2N as well as the weakly tumorigenic S2 cells exhibit similar cell morphologies when grown as 2D monolayer or as 3D culture in suspension (Fig. 2). The 3D morphologies resemble tight aggregates rather than true spheres, which obviously is due to the absence of E-cadherin in both cell models (28). For any phenotype analysis and functional *in vitro* study, cells were cultivated to subconfluency (monolayer) or to 3D sizes (spheres) as shown in Fig. 2.

Since *in vitro* growth rate differences might affect comparative *in vivo* cell analytical results, we evaluated whether *in vitro* cell cycle differences exist between serum-free cultivated spheres and the monolayer cells grown in 10% FBS. To this end, we applied a high resolution 48 h cell cycle kinetic technique that displays up to 3 consecutive cell cycles within one sample (29). Fig. 2 shows that the S2N monolayer cultures grew somewhat faster since more cells have already reached the G2M’ phase in the 2nd and the G1” phase in the 3rd cell cycle compared to the sphere culture. The media composition did not affect the cell growth rate since there was no difference in the cell cycle kinetic pattern. The cell cycle kinetic pattern of weakly tumorigenic S2 cells was neither affected by different medium serum composition nor by 2D or 3D cultivation technique (lower panels Fig. 2).

### Monolayer cells display a differentiation-like phenotype

**Figure 3:**
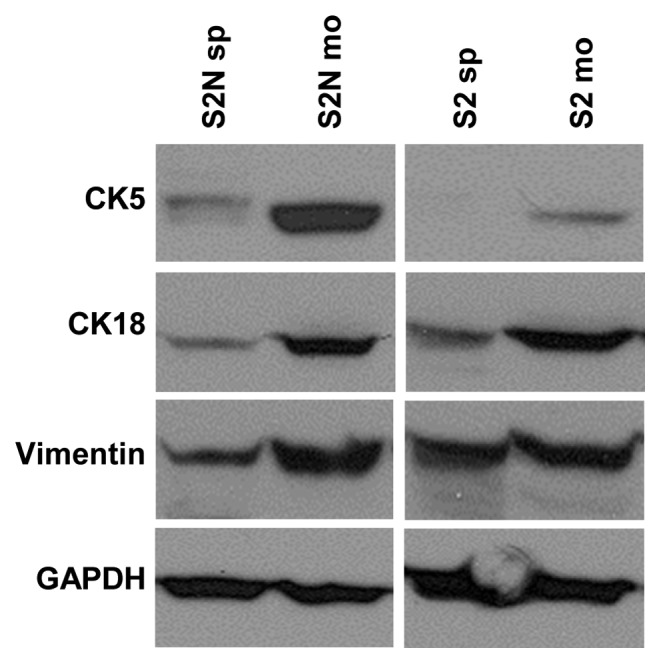
Western blot analysis of intermediate filament expression in highly tumorigenic S2N sphere cells compared to weakly tumorigenic, more differentiated S2N monolayer cells, S2 sphere and S2 monolayer cells. Breast cancer cell differentiation markers: cytokeratin 5 (basal breast cancer) and cytokeratin 18 (luminal breast cancer), vimentin (mesenchymal marker) and GAPDH loading control.

To determine the differentiation status of monolayer and sphere cells, we performed western blot analyses targeting the epithelial markers cytokeratin 5 (basal) and 18 (luminal) as well as the mesenchymal marker vimentin (Fig. 3). 2D monolayer cultures were derived from spheres by transferring the cells from a serum-free environment to serum-containing medium. Cells were allowed to adhere and grow on a collagen-coated surface for at least 10 days. Differentiation-like events are indicated by upregulation of epithelial markers. Indeed, basal cytokeratin 5 is slightly expressed in monolayer cells of line S2, whereas no CK5 was detected in corresponding spheres. Strong CK5 upregulation was identified in S2N monolayers. Differences in luminal cytokeratin 18 expression were similar for both cell lines with slight upregulation in monolayer cells. Furthermore, the decrease of cadherin-11 (change factor 99.7), N-cadherin (16.3), SnaI2 (0.6) and Twist (0.8) mRNA in S2N monolayer cells indicates a more differentiated status of monolayer cells compared to serum-free cultured spheres. This suggests that the spheroid cells might have undergone epithelial-to-mesenchymal-transition (EMT) processes. The intermediate filament marker vimentin was not useful as mesenchymal indicator, since it is expressed in both cell lines independently of tumorigenicity in the mouse xenograft model.

### Established cancer stem cell markers are not indicative of in vivo tumorigenicity

**Figure 4:**
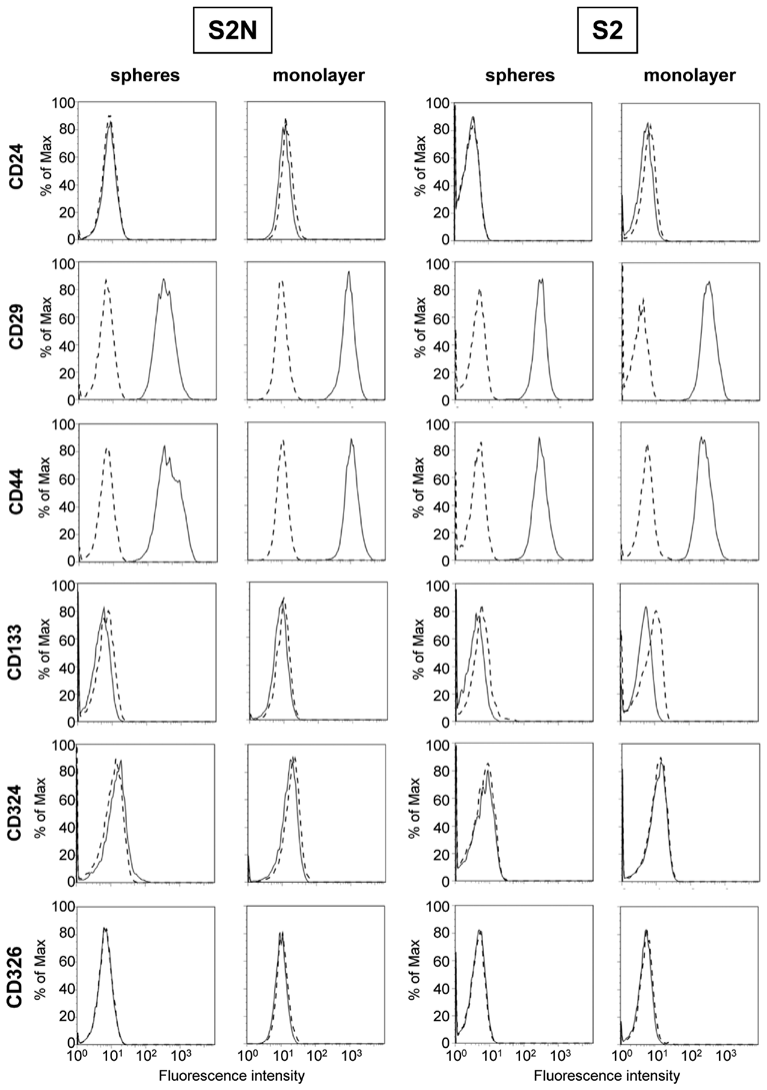
FACS expression analysis of putative CSC/TIC cell surface receptors of highly tumorigenic S2N sphere cells compared to more differentiated, weakly tumorigenic S2N monolayer cells, S2 sphere and S2 monolayer cells. CD324 marker, E-cadherin; CD326 marker, EpCAM. FACS receptor analysis was done by gating on viable, non-aggregated single cells. IgG isotype control (broken line) and receptor specific antibody (solid line).

In order to verify published cancer stem cell markers, we first compared surface molecule expression on monolayer and sphere cells and evaluated differential expression on highly tumorigenic cell line S2N and weakly tumorigenic line S2 (Fig. 4). All antibodies used were verified using appropriate cell lines (data not shown). As displayed in Fig. 4, both S2 and S2N lines are CD24 negative, but strongly express CD44. There is no difference in the CD24/CD44 expression pattern, either between spheres and monolayer of the same cell line, or in comparison of the highly tumorigenic versus the weakly tumorigenic cell line. Moreover, CD24 and CD44 double staining dot plots did not reveal any distinct, minor subpopulations (data not shown). Similar to CD44, we confirmed the presence of putative cancer stem cell marker CD29, but the expression on sphere and mono-layer cells of both cell lines was almost identical. The measured fluorescence intensity of bound CD29 antibody was roughly two decades above background on all sphere and monolayer cells. In addition to the CD24/CD44 marker set, CD29 expression is also not correlated with the aggressive growth characteristics of highly tumorigenic S2N spheres in mice. Moreover, established cancer stem cell markers CD133 and CD326 (EpCAM) were not detected on any cell line (Fig. 4). Loss of CD324 (E-cadherin) is thought to indicate an EMT process. All cell types were found to be E-cadherin negative and therefore this marker cannot be correlated with *in vivo* growth characteristics of highly and weakly tumorigenic cells.

**Figure 5:**
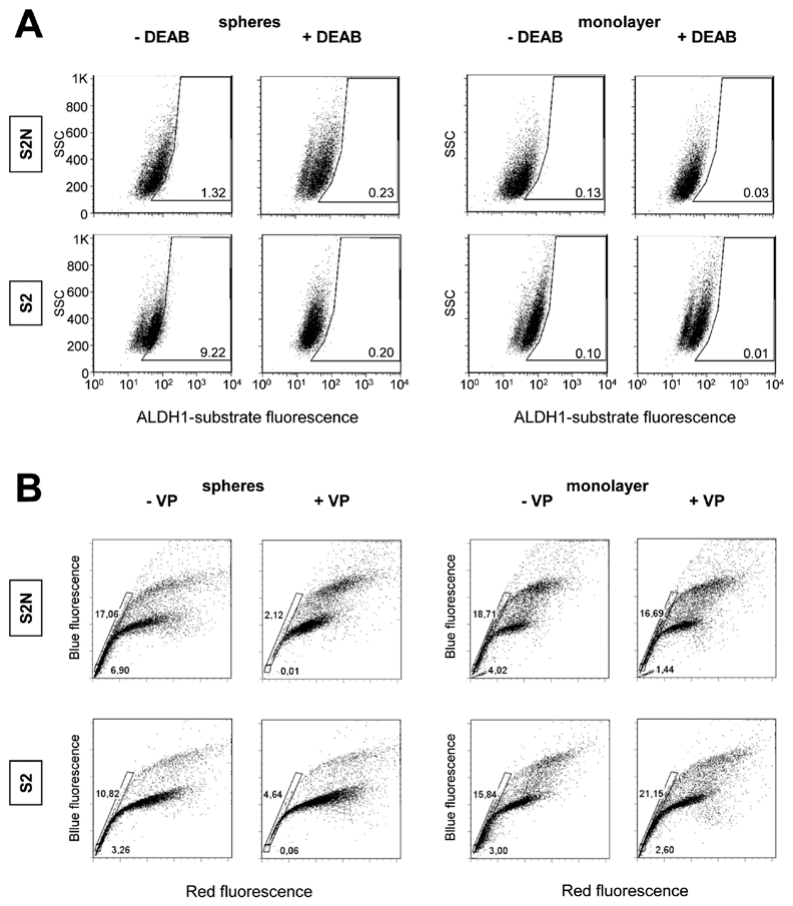
Aldehyde dehydrogenase 1 (ALDH1) and Hoechst side population (SP) activity in S2N and S2 sphere and monolayer cells. (A) ALDH1 activity in absence or presence of the ALDH1 inhibitor DEAB. Cells within framed region represent ALDH1 positive cells. (B) Multidrug resistance transporter activity in absence or presence of MDR transporter inhibitor verapamil. The framed region indicates cells of the Hoechst 33342 side population (cellular fluorochrome exclusion). FACS gating in both assays was on viable, non-aggregated single cells.

Increased ALDH1 activity has been reported as a marker for cancer stem or stem-like cancer cells of some human malignancies. The functionality of the ALDH1 assay was validated by the DEAB sensitive, CD34^+^/CD38^−^ stem/early progenitor cell fraction from human bone marrow (data not shown). We then tested S2N and S2 sphere and monolayer cells for ALDH1 activity. A baseline fluorescence region was set close to the cluster of DEAB treated cells (Fig. 5A). Fig. 5, which displays a representative experiment out of 3 replicates, shows that independent of the *in vivo* tumorigenicity, S2N and S2 sphere cells exhibited a fraction of DEAB sensitive ALDH1^+^ cells. Interestingly, this subpopulation was even higher in the weakly tumorigenic S2 (± DEAB, 9.22/0.2%) compared to the highly tumorigenic S2N cells (± DEAB, 1.32/0.23%). Much smaller fractions of DEAB-sensitive ALDH1 positive cells were found in the monolayer cells from both S2N and S2 cells. These findings are supported by our gene array study which revealed that none of the ALDH1 isoforms 1A1, 1A2, 1A3, 1B1, 1L1 and 1L2 showed any correlation with the tumorigenicity of the cells (data not shown). Of note is the fact that the cell clusters shift to smaller ALDH1 values in the presence of the DEAB with no evidence of a tumorigenicity related change (fluorescence means ± DEAB, S2 spheres 46.9/24.3; S2 monolayer 68.1/51.0; S2N spheres 61.3/45.4; S2N monolayer 32.9/34.8). Finally, our analyses indicate the absence of true ALDH1 positive subpopulations (Fig. 5A).

SP cells were reported to express various stem cell markers, exhibit self-renewal capabilities and generate differentiated cells. We investigated a putative association between SP incidence in the cell lines and their tumorigenicity in mouse xenograft experiments utilizing flow cytometry and Hoechst dye efflux assays. As cells discard Hoechst 33342, a discrete side population is formed in a Hoechst blue/Hoechst red plot, appearing at the lower left to the stained cell clusters of G1, S and G2 phases (Fig. 5B). The ABCG2 inhibitor verapamil indicates the specificity of the SP relevant transporter. Spheres of highly tumorigenic line S2N as well as spheres of weakly tumorigenic cells display verapamil sensitive side populations (Fig. 5B). S2N spheres contain a slightly higher percentage of SP cells (18%, with VP 2%) than S2 spheres (11%, with VP 5%). Interestingly, all monolayer cells exhibit an even higher portion of SP cells (S2N, 19%; S2, 17%), but dye efflux was not reduced by adding verapamil. The expression analysis of Hoechst 33342 transporter ABCG2 also showed no correlation with the tumorigenicity of the cells (S2N spheres 13.5, S2 spheres 236.4, S2N monolayer 90.6, S2 monolayer 176.9).

### Highly tumorigenic S2N spheres display a distinct mRNA expression pattern compared to monolayer cells and weakly tumorigenic S2 spheres

**Figure 6:**
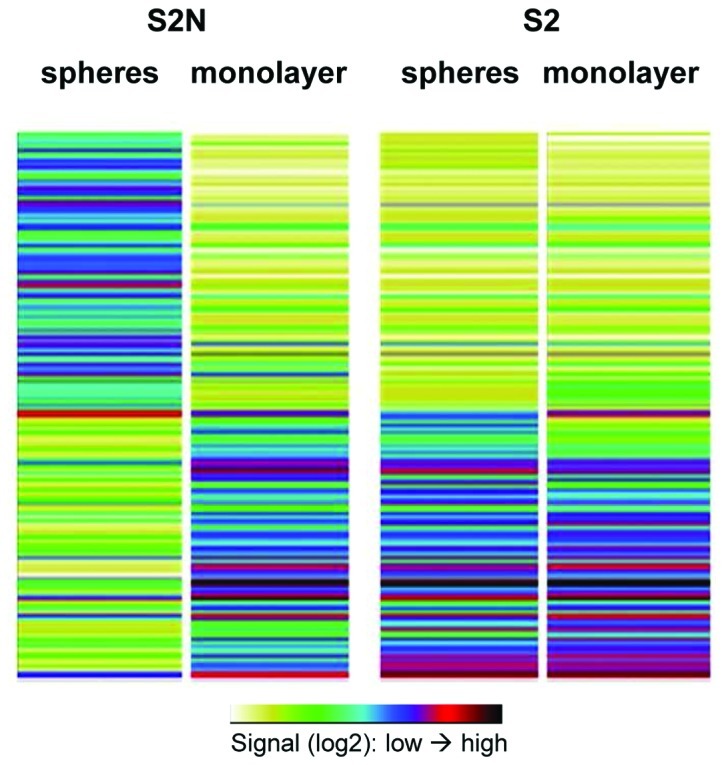
Comparison of mRNA gene expression pattern of highly tumorigenic S2N sphere cells, more differentiated weakly tumorigenic S2N monolayer cells and weakly tumorigenic S2 sphere and monolayer cells. The comparison is based on 183 probe sets representing 136 genes which are up- or downregulated differently by at least a factor of 2.

Comparison of the gene expression patterns of S2N and S2 sphere cells to their respective monolayer counterparts revealed a unique expression pattern of highly tumorigenic S2N sphere cells (Fig. 6). Furthermore, there was a high similarity between the weakly tumorigenic S2 sphere and monolayer cells with the more differentiated S2N monolayer cells indicating that the *in vitro* cultivation technique cannot solely explain the tumorigenicity of our cell models.

To identify transcription factors reported to be relevant for normal and tumor stem cell phenotype, we performed an in depth gene expression analysis of the highly tumorigenic S2N sphere versus their more differentiated, weakly tumorigenic monolayer daughter cells. Stem cell associated mRNA species such as Oct3, Sox1, Sox2 or Nanog were undetectable in both cell lines (Table I). Although Oct3 and Sox1 proteins were weakly expressed and detectable by western blot analysis, there was no increased expression in the highly tumorigenic S2N spheroid cells (data not shown). Except KLF4 and Notch 3, most of the stem cell relevant transcription factors were even expressed at higher levels in monolayer cells. A slightly higher expression of both EMT transcription factors SnaI2 and Twist1 was found in the highly tumorigenic sphere cells, but the expression differences were rather small.

**Figure 7:**
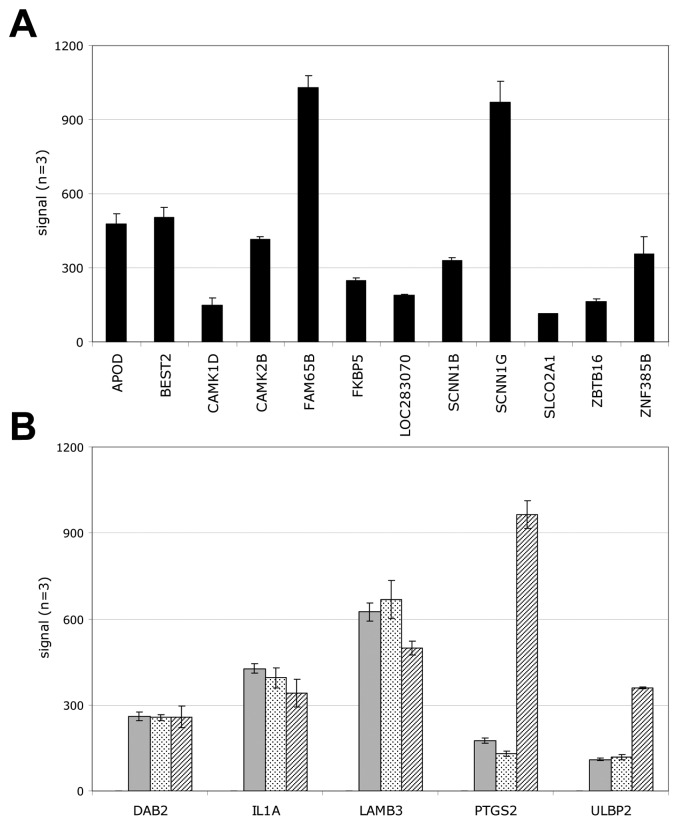
Genes exclusively expressed in highly tumorigenic or weakly tumorigenic cells. (A) Genes expressed in the highly tumorigenic S2N spheroids only but not in weakly tumorigenic S2 spheroid/monolayer and S2N monolayer cells. (B) Genes expressed in weakly tumorigenic S2 spheroid/monolayer and S2N monolayer cells but not expressed in highly tumorigenic S2N spheroids (filled bars, S2 spheroids; dotted bars, S2N monolayer; hatched bars, S2 monolayer).

To identify genes that may explain the difference in tumorigenicity of our cell models, we screened our array data for genes which were either exclusively expressed in tumorigenic S2N spheroid cells or for transcripts that were completely absent. Using these filter criteria, we additionally eliminated gene expression differences due to the different cell culture formats. Fig. 7 shows that 12 genes were expressed solely in the highly tumorigenic S2N cells and 5 genes were not. The exclusively expressed genes include the EMT inducer CAMK1D, the stem cell maintenance gene ZBTB16, tumorigenicity related FAM65B and FKBP5 as well as an invasion facilitating non-voltage Na^+^ channel SCNN1G (Fig. 7A). Genes not expressed in the highly tumorigenic cells encompass the EMT process inhibiting DAB2 and LAMB3 which is frequently inactivated in breast cancer cells increasing their invasiveness (Fig. 7B). Interestingly, two mRNAs relevant for immune escape are also absent in the tumorigenic cells (IL1A, ULBP2).

## Discussion

In a variety of ontogenetic different tumors, tumor-initiating cells have been identified. These cells exhibit more aggressive *in vivo* growth characteristics, and increase the complexity of *in vivo* tumors (3,7). However, there is not only controversy on the hierarchical versus clonal evolution of putative TICs but also on the molecular, physiological and phenotypic markers to identify these cells (5,6,13). In breast cancer, the CD24^−^/low/CD44^+^ marker combination has been shown to identify *in vivo* tumorigenic cell subpopulations (10), although the percentage of the subfraction did not correlate with the *in vivo* tumorigenicity (11). Furthermore, the presence of CD24^−^/CD44^+^ or CD24^+^/CD44^+^ cells in primary tumors did not correlate with the overall or metastasis-free survival of breast cancer patients (21).

Representative data from our S2N and S2 cell lines also challenge the validity of the CD24^−^/CD44^+^ marker combination and EpCAM expression as tumorigenicity markers in breast cancer. All sphere and monolayer derivative cell models investigated in this report exhibit a unique CD24^−^/CD44^+^ marker distribution, although only the S2N spheres were highly tumorigenic at low inoculation cell numbers *in vivo* (Figs. 1 and 4; confirmed in 2 more primary breast cancer cell models, data not shown). The highly tumorigenic as well as the weakly tumorigenic cell models were EpCAM negative and profiling analysis revealed a normal-like to basal-like breast cancer origin (30). EpCAM negative breast tumor spheres have been generated from primary tumors (27), and EpCAM negative, immortalized epithelial breast cancer stem cells have been reported previously (Weinberg R, EMT and Cancer Progression Meeting, Arlington, VA, 2010). Finally, basal-like and normal-like breast cancer cell lines with a mesenchymal EMT phenotype such as MDA-MB 468 and MDA-MB 231, respectively, also lack EpCAM expression (31). Therefore, EpCAM expression should not be considered as an *in vivo* tumorigenicity marker for normal-like to basal-like breast cancer stem cells (10).

Furthermore, there was no difference in physiological CSC markers between our highly tumorigenic and weakly tumorigenic cell models. The validation experiment of ALDH1 activity in early hematopoietic CD34^+^/CD38^−^ cells showed a clear DEAB inhibition (data not shown). However, we did not identify an ALDH1 positive, DEAB sensitive subpopulation indicative of a more tumorigenic cell fraction in any cell line tested (Fig. 5A). This is in contrast to reports showing that sorted ALDH1 positive breast tumor cells from primary tumors (19) or established cell lines (20,21) are highly tumorigenic compared to their ALDH1 negative counterparts. However, in a recent breast cancer IHC study, the cumulative patient survival rate was not different between ALDH1 positive and negative tumors (32). Additionally, 70 to 80 or even 93% of the breast tumor specimens proved to be negative for ALDH1 expression (19,32). Apparently, our ALDH1 negative, primary cancer derived, highly tumorigenic mammosphere breast cancer cell line represents such an ALDH1 negative CSC/TIC population.

In line with our ALDH1 results is the absence of a specific verapamil sensitive side population (SP) in the highly tumorigenic cell model. A large verapamil sensitive SP fraction was identified in highly tumorigenic S2N spheres as well as in weakly tumorigenic S2 spheres (Fig. 5B). Furthermore, even slightly higher percentages of SP were present in both weakly tumorigenic monolayer counterparts, although there was no or little verapamil sensitivity. Therefore, the *in vivo* tumorigenicity is obviously not correlated with the verapamil sensitive SP and one is tempting to speculate that the major role of this cell fraction might just be a higher resistance to exogenous toxic exposures (16,22).

The increase of basal (CK5) and luminal (CK18) cytokeratins in both monolayer TIC cell lines are indicative of a mesenchymal-epithelial transition, differentiation-like event (Fig. 3). Since there is no correlation with the *in vivo* phenotype, it could also be possible that the cytokeratin MET pattern alteration is a surrogate marker only. This hypothesis is supported by the fact that vimentin is expressed at almost equal levels in the mammosphere as well as in the monolayer cells. Finally, although the absence of E-cadherin and the expression of vimentin should indicate a more mesenchymal, de-differentiated phenotype (4,23), our weakly tumorigenic S2 cell line puts the validity of these markers as tumorigenicity identifier into question. Our suggestion is supported by the fact that, in contrast to recent reports (24,25), the induction of a more mesenchymal cell phenotype increases the transcription factor mRNAs of SnaI2 and Twist1 in breast cancer cells only by a factor of 1.6 and 1.8, respectively (Table I). Therefore, it is unlikely that both factors solely mediate a more aggressive, tumorigenic mesenchymal phenotype *in vivo*.

None of the stem cell related transcription factors display a unique TIC expression pattern (Table I). *Oct3*, *Sox1/2* or *Nanog* mRNAs are undetectable and *myc* is not changed in the highly tumorigenic S2N cell line. The Sox transcription factors are even upregulated in the weakly tumorigenic monolayer cells and only *KLF4* and *Notch3* are upregulated in the S2N mammospheres. Finally, an 11-gene signature previously reported to be predictive for therapy failure in multiple cancers (33) was not predictive for the tumorigenicity of our cell models.

Our gene array data indicate that the molecular mechanisms affecting the tumorigenicity of tumor-initiating cells extend beyond the established markers. Genes that are only expressed in our tumorigenic breast cancer cells encompass the EMT inducer CAMK1D (34), the stem cell maintenance gene ZBTB16 (=PLZF) (35) as well as the tumorigenicity related FKBP5 (36). Not only genes that are upregulated or exclusively expressed in tumorigenic spheres, but also genes that are down-regulated or not expressed, such as the EMT process inhibiting DAB2 (37) and the invasiveness lowering LAMB3 (38), may contribute to the tumorigenicity of our models (Fig. 7).

In addition to intrinsic, genetically regulated pathways, environmental factors affect the physiology and phenotype of tumor cells (39,40). Inoculation of high monolayer or sphere cell numbers of S2 cells displayed similar *in vivo* growth characteristics compared to the highly tumorigenic TICs, whereas at low cell numbers only S2N spheres were highly tumorigenic (10,19). Currently, the most common explanation for this ‘low sphere cell number’ phenomenon is the higher percentage of putative TICs in the tumorigenic sphere cell population. However, if TICs exhibit a more aggressive growth phenotype, why is there no higher growth rate at higher *in vivo* cell inoculation numbers? An alternative explanation for the growth difference might be an improved adaptive survival response at low tumor inoculation cell numbers of the tumorigenic mammosphere breast cancer cells *in vivo*, which was shown previously by the flexibility of TIC marker expression during *in vivo* passages (14). Further support comes from our findings that our gene chip array analysis did not reveal a unique, tumorigenicity related alteration of a cyclin/CDK or anti-apoptotic gene expression pattern (Fig. 7).

In summary, we give experimental evidence that established cancer stem cell markers do not correlate with *in vivo* growth characteristics of tumor-initiating cells. We show that highly tumorigenic as well as weakly tumorigenic sphere cell lines and their monolayer derivatives do not exhibit any difference in putative TIC specific CD markers, ALDH1 activity or SP fraction. Stem cell related transcription factors are not increased in the highly tumorigenic cell line. Therefore, we suggest that other functional, still unknown markers and/or environmental factors might affect the increased tumorigenicity of breast cancer sphere cell lines.

## Figures and Tables

**Table I:** Expression of stem cell relevant transcription factors in S2N cells.

Gene name	Locus ID	Change expression monolayer vs. sphere	Remark
*Oct3* (*Oct 4*)	5460		No signal above background
*Sox1*	6656		No signal above background
*Sox2*	6657		No signal above background
*Sox4*	6659	2.74	
*Sox7*	83595	1.22	
*Sox9*	6652	1.35	
*KLF4*	9314	−1.36	
*Notch1*	4851	0.23	Statistically not significant
*Notch2*	4853	0.67	
*Notch3*	4854	−3.30	
*Notch4*	4855	0.24	Statistically not significant
*Nanog*	79923		No signal above background
*Myc*	4609	0	
*SnaI2* (*Slug*)	6591	−0.59	
*Twist1*	7291	−0.83	

A positive value of the change factor indicates higher mRNA expression in monolayer cells.
